# Comparison of the pathological outcome and disease progression of two *Mycobacterium caprae* experimental challenge models in goats: endobronchial inoculation vs. intranasal nebulization

**DOI:** 10.3389/fmicb.2023.1236834

**Published:** 2023-08-11

**Authors:** Cristian Melgarejo, Alex Cobos, Carles Planas, Jaume Fondevila, Maite Martín, Zoraida Cervera, Guillermo Cantero, Xavier Moll, Yvonne Espada, Mariano Domingo, Enric Vidal, Bernat Pérez de Val

**Affiliations:** ^1^Unitat Mixta d'Investigació IRTA-UAB en Sanitat Animal, Centre de Recerca en Sanitat Animal, Campus de la Universitat Autònoma de Barcelona, Bellaterra, Spain; ^2^IRTA, Programa de Sanitat Animal, Centre de Recerca en Sanitat Animal, Campus de la Universitat Autònoma de Barcelona, Bellaterra, Spain; ^3^Departament de Sanitat i Anatomia Animals, Universitat Autònoma de Barcelona, Bellaterra, Spain; ^4^Fundació Hospital Clínic Veterinari, Universitat Autònoma de Barcelona, Bellaterra, Spain; ^5^Departament de Medicina y Cirugía Animals, Universitat Autònoma de Barcelona, Bellaterra, Spain

**Keywords:** tuberculosis, *Mycobacterium caprae*, goat, experimental infection, intranasal, endobronchial

## Abstract

**Background:**

Goats are natural hosts of tuberculosis (TB) and are a valid animal model to test new vaccines and treatments to control this disease. In this study, a new experimental model of TB in goats based on the intranasal nebulization of *Mycobacterium caprae* was assessed in comparison with the endobronchial route of infection.

**Methods:**

Fourteen animals were divided into two groups of seven and challenged through the endobronchial (EB) and intranasal (IN) routes, respectively. Clinical signs, rectal temperature, body weight, and immunological responses from blood samples were followed up throughout the experiment. All goats were euthanized at 9 weeks post-challenge. Gross pathological examination, analysis of lung lesions using computed tomography, and bacterial load quantification in pulmonary lymph nodes (LNs) by qPCR were carried out.

**Results:**

The IN-challenged group showed a slower progression of the infection: delayed clinical signs (body weight gain reduction, peak of temperature, and apparition of other TB signs) and delayed immunological responses (IFN-γ peak response and seroconversion). At the end of the experiment, the IN group also showed significantly lower severity and dissemination of lung lesions, lower mycobacterial DNA load and volume of lesions in pulmonary LN, and higher involvement of the nasopharyngeal cavity and volume of the lesions in the retropharyngeal LN.

**Conclusion:**

The results indicated that the IN challenge with *M. caprae* induced pathological features of natural TB in the lungs, respiratory LN, and extrapulmonary organs but extremely exaggerating the nasopharyngeal TB pathological features. On the other hand, the EB route oversized and accelerated the pulmonary TB lesion progression. Our results highlight the need to refine the inoculation routes in the interest of faithfully reproducing the natural TB infection when evaluating new vaccines or treatments against the disease.

## 1. Introduction

Tuberculosis (TB) is an infectious disease caused by mycobacteria belonging to the *Mycobacterium tuberculosis* complex (MTBC) that affects humans and a wide range of domestic animals and wildlife. TB in domestic goats is mainly caused by *Mycobacterium caprae*, a member of the MTBC (Aranaz et al., 2003). The disease has an economic impact resulting from production losses (Seva et al., 2002; Daniel et al., 2009), and infected goats pose a risk of infection to other animal species (Napp et al., 2013; Cano-Terriza et al., 2018; Vidal et al., 2018) and humans (Rodríguez et al., 2011).

TB in goats is usually transmitted through the aerogenous route, and it is common to find lesions mainly in the respiratory tract (Domingo et al., 2014). Goats develop caseous-necrotizing cavitary lesions similar to those observed in human TB (Pérez de Val et al., 2011; Sanchez et al., 2011), whereas these lesions are rarely developed in cattle (Buddle et al., 2016). Different approaches exist to control livestock TB, and the test and slaughter strategy is the most widely implemented, particularly in cattle. Vaccination against TB could be considered as an alternative approach. However, cattle vaccination against TB is currently forbidden in the EU (Council Directive 78/52/EEC) due to interference with currently used TB diagnostic tools. The goat model of TB has proven to be a suitable one to study new TB vaccines for ruminants and humans and for a better understanding of the pathogenesis of the disease (Ramirez et al., 2003; Pérez de Val et al., 2011, 2013; Wedlich et al., 2022).

In the different TB studies using the goat model, the infection has typically been carried out through the endobronchial route (Pérez de Val et al., 2011, 2012, 2013; Arrieta-Villegas et al., 2018, 2020; Melgarejo et al., 2022), intratracheal aerosolization (Gonzalez-Juarrero et al., 2013), and transthoracic injection (Bezos et al., 2010, 2015), all of them inducing primarily pulmonary TB lesions. In particular, the endobronchial approach allows for an accelerated progression of pulmonary TB, which is positive in terms of reducing the duration of the experiments. However, if the infectious dose is not well-adjusted, exaggeratedly large lung lesions may be produced. These large lesions do not only differ from the common natural presentation of the disease but also might preclude a proper evaluation of the vaccine effect (Arrieta-Villegas et al., 2020). A completely different experimental approach is the “natural” infection by long-term direct-contact exposure of experimental goats with infected animals in controlled conditions (Bezos et al., 2017; Roy et al., 2018, 2019). The advantage of this approach is that it induces all the natural features of the infection, but other aspects such as infection dose or infection time point remain uncontrolled, and it dramatically increases the duration of the experiment. Nowadays, *in vitro/ex vivo* and *in silico* experimental models are being developed to replace or reduce animal models in preclinical studies of vaccine candidates against TB (Tanner et al., 2016; Català et al., 2020). However, the optimization of these approaches requires a comparison to well-characterized animal models that can be used as “gold standard”. Altogether, an accurate standardization of a goat model of TB is critical to predicting the efficacy of new vaccine candidates. This study aimed to evaluate and characterize the experimental infection of goats with *M. caprae* by intranasal nebulization in comparison with the endobronchial route. The rationale was to use a more natural access path of mycobacteria to the respiratory tract than the endobronchial route to reproduce more precisely the spectrum of lesions of goat TB found in field cases.

## 2. Materials and methods

### 2.1. Animals and experimental infections

Fourteen 3–4 months old female Pyrenean goats, from an officially tuberculosis-free herd, located in the Catalan Pyrenees (Spain), were chosen for this study. Experimental animals were transferred to the IRTA-CReSA Biosafety Level 3 (BSL-3) facility (Catalonia, Spain), where they were weighted and randomly divided into two experimental groups of seven animals each, challenged by the endobronchial (EB) and intranasal (IN) routes, respectively. According to body weights, corrections to ensure homogenous distribution between the groups were applied, and then, the animals were housed in two experimental boxes with animals 4–3 and 3–4 from the EB and IN groups in each box, respectively. The animals were fed hay, alfalfa, feed, and mineral salt and had access to water *ad libitum* throughout the experiment.

After 1 week of acclimatization, the animals were sedated intramuscularly with acepromazine maleate (0.05 mg/kg) and butorphanol (0.2 mg/kg) and subsequently anesthetized with intravenous administration of propofol (5 mg/kg) and midazolam (0.2 mg/kg). Afterward, the animals were challenged with 0.5 ml of a *M. caprae* field strain (Balseiro et al., 2017) at a suspension of 2 × 10^3^ CFU/ml. Animals of the EB group were challenged through the endobronchial route as previously described (Pérez de Val et al., 2011).

The animals of the IN group were challenged by using a syringe-adapted device for atomized spraying (MADgic^®^, Wolfe Tory Medical, Inc., Salt Lake City, USA). This device consists of a flexible cannula measuring 21.6 cm in length and 4.82 mm in width with a nebulizer located at its end that generates droplets of 30–100 μm in diameter, according to the manufacturer's instructions. The cannula was inserted into the nasal cavity ~10–15 cm depending on the size of the animal. Each side of the nasal cavity received 0.5 ml of the same *M. caprae* strain at a suspension of 10^3^ CFU/ml without anesthesia.

After the challenge, all animals were daily monitored for clinical signs by a veterinarian to assess whether any of the animals reached the endpoint criteria. The clinical evaluation included 0–3 scoring of each of the following five parameters: body condition, mental state/behavior, weight loss, fever, and the presence of respiratory signs (0: absence of respiratory signs; 1: mild dyspnea and/or occasional cough; 2: moderate dyspnea and continued coughing; and 3: marked dyspnea, continued coughing, and/or severe nasal discharge). Any global score higher than 11 and/or the presence of qualitative criteria such as prostration, lack of movement, or severe respiratory distress resulted in euthanasia. Rectal temperature and body weight were measured each week. Heparinized blood samples were collected at weeks 0 (before challenge), 3, 5, 7, and 9 (endpoint) for immunological assays.

### 2.2. Ethics statement

All the procedures with experimental animals carried out during the study were approved by the Animal Welfare Committee of the *Generalitat de Catalunya* (Project Ref. #10794), in accordance with the European Union legislation for the protection of experimental animals (86/609/CEE, 91/628/EEC, 92/65/EEC, and 90/425/EEC).

### 2.3. Antigens and reagents

*M. bovis* tuberculin (PPD-B, 2,500 IU/ml) was obtained from CZ vaccines (Porriño, Spain). The specific recombinant antigens of MTBC ESAT-6, CFP-10, and MPB83 were obtained from Lionex (Braunschweig, Germany) at a concentration of 500 μg/ml each. ESAT-6 and CFP-10 were mixed 1:1 in an antigenic (E/C) cocktail. The P22 antigenic complex was produced by immunopurification of PPD-B (CZ vaccines) as described previously (Infantes-Lorenzo et al., 2017) and was supplied by the *Carlos III Research Institute* (Madrid, Spain) at a concentration of 500 μg/ml.

### 2.4. Whole blood IFN-γ release assay

Approximately 10 ml of whole blood was collected at the abovementioned time points from the jugular vein using heparinized tubes. Blood samples were maintained at room temperature for <1 h when they were stimulated in 96-well cell culture plates (Eppendorf, Hamburg, Germany) with PPD-B, P22 (except at week 7), and E/C at a final concentration of 20 μg/ml each. Phosphate-buffered saline was used as an unstimulated control. The samples were incubated at 37°C and 5% CO_2_ for 18 ± 2 h. Plasma supernatants were collected after centrifugation at 18 *g* for 10 min, and the released IFN-γ was measured by ELISA (ID Screen^®^ Ruminant IFN-g kit, ID.vet, Grabels, France). Optical densities of the ELISA plates were read at 450 nm (OD_450nm_) using a spectrophotometer (Biotek Power Wave XS^®^, Agilent, Santa Clara, USA). Antigen-specific IFN-γ responses were calculated as OD_450nm_ of the antigen-stimulated well minus OD_450nm_ of the unstimulated well (ΔOD_450nm_).

### 2.5. Antibody detection assays

Plasma samples from all experimental animals were analyzed in duplicate to follow antibody responses against MTBC after the challenge. Indirect ELISAs were used to detect total IgG against MPB83 antigen and P22 antigenic complex, respectively. The two ELISAs were performed as previously described (Pérez de Val et al., 2017; Infantes-Lorenzo et al., 2019). MPB83-IgG levels were calculated as OD_450nm_ of the antigen-coated well minus OD_450nm_ of the uncoated well (ΔOD_450nm_). A sample was considered positive when ΔOD_450nm_ ≥ 0.05. P22-IgG levels were calculated as a percentage of ELISA (E%) = [mean OD_450nm_ of the antigen-coated well/(2 × mean negative control OD_450nm_)] × 100. A sample was considered negative when E% ≤ 100%, non-conclusive when E% was between 110 and 150%, and positive (seroconversion) when E% ≥ 150%.

### 2.6. Necropsy, pathological examination, and computed tomography

At week 9 post-challenge, all goats were sedated intramuscularly by an injection of acepromazine maleate (0.1 mg/kg) and butorphanol (0.2 mg/kg) and subsequently euthanized with an intravenous overdose of sodium pentobarbital. At necropsy, the size of TB-compatible lesions in the retropharyngeal (right and left), mediastinal (cranial and caudal), and tracheobronchial (LN) lymph nodes was measured, and the volume of lesions was inferred using the volume formula of the most similar 3D-geometrical morphology (including sphere, cylinder, or prism), as previously described (Balseiro et al., 2017). Other visible lesions in extrapulmonary tissues were also recorded and fixed in 10% buffered formalin to be confirmed by histopathology and Ziehl–Neelsen staining to detect acid-resistant bacilli. The lungs were fixed by airway instillation followed by immersion in formalin, as previously described (Pérez de Val et al., 2011), and then scanned by computed tomography (CT) using a 16-slice multidetector scanner (Brivo CT-385, General Electric Healthcare, Madrid, Spain). CT analysis was performed as previously described (Balseiro et al., 2017). In brief, total volumes of the lungs and TB lesions were measured using volume representation images with different density patterns (i.e., solid, mineralized, and cavitary lesions), and the total volume of mineralization was calculated using 100–300 Hounsfield units.

### 2.7. Quantification of bacterial DNA

After macroscopic pathological evaluation, mediastinal and tracheobronchial LNs were collected and stored at −20°C until they were processed for bacteriology. The whole LNs were then thawed and homogenized in 10 ml of sterile-distilled water using a homogenizer (Masticator, IUL Instruments, Barcelona, Spain). An aliquot of 1 ml of each homogenate was inactivated at 75°C for 1 h for bacterial DNA quantification. In parallel, an aliquot of 100 ml of the master seed *of M. caprae* (~10^8^ CFU/ml), used for the challenged inoculum, was inactivated and then diluted in eight 10-fold dilutions to establish the standard curve. DNA samples were extracted using an ID Gene™ spin universal extraction kit (ID.vet) and amplified with the *Mycobacterium tuberculosis* complex duplex kit (ID.vet), following the manufacturer's instructions. The amplification was performed in a 7500 fast real-time PCR system (Applied Biosystems, Waltham, MA, USA). The genomic equivalents of CFU were calculated as previously described (Vidal et al., 2022).

### 2.8. Data analysis

A completely randomized design was performed to compare the effects of *M. caprae* infection (primary factor) by the EB and IN routes. First, all data distributions were analyzed by using the Shapiro–Wilk normality test. Temperature and body weight were compared using a unidirectional unpaired *t*-test. Antigen-specific immune responses (measured by IGRA, IgG-ELISA), lesion volumes, and mycobacterial DNA load were compared using the non-parametric Mann–Whitney test. GraphPad Prism version 8.0.0 (San Diego, CA, USA) was used for statistical analyses.

## 3. Results

### 3.1. Endobronchial challenge induced earlier and stronger immune responses

**Figure 1:**
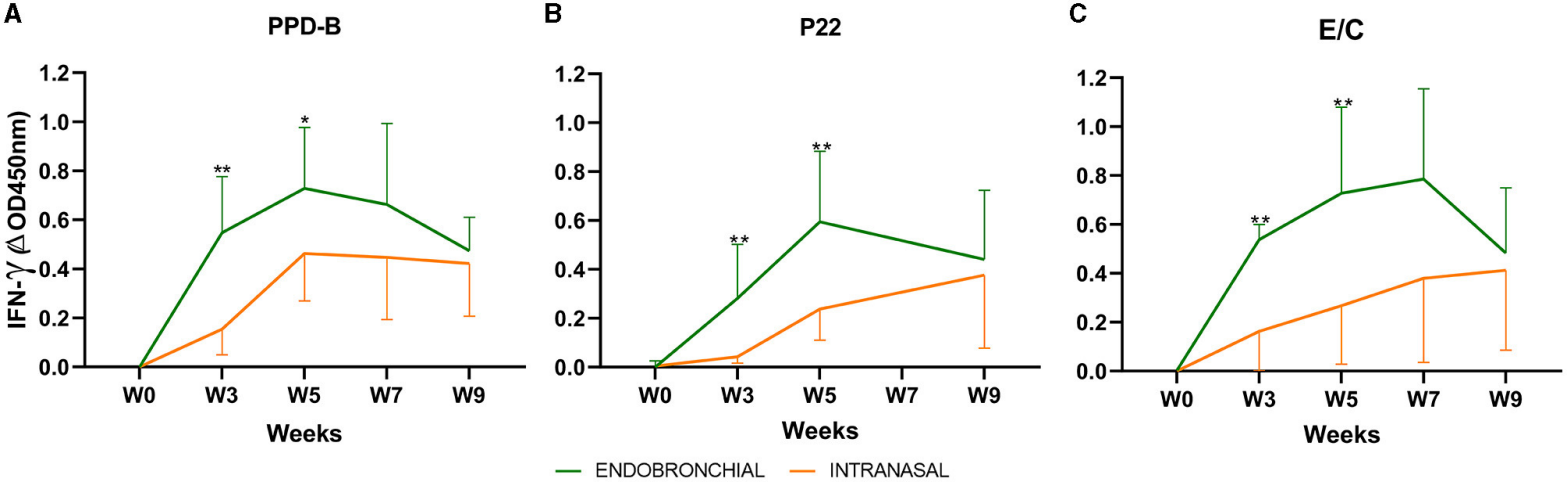
Whole blood IFN-γ responses before and after challenge: the graph shows the IFN-γ levels measured by ELISA. The results are expressed as ΔOD450mn ± 95% CI. **(A)** Response against bovine tuberculin (PPD-B). **(B)** Response to the P22 complex. **(C)** Response against E/C antigen cocktail. *P < 0.05, **P < 0.01, Mann–Whitney test. Groups: Endobronchial (*n* = 7, green) and Intranasal (*n* = 7, orange). Two animals of the endobronchial group were humanely sacrificed at week 7 and one animal of the group Intranasal.

Mean antigen-specific IFN-γ responses in whole blood before and after the challenge for each treatment group are presented in Figure 1. An increase in IFN-γ-specific responses to PPD-B, P22, and E/C antigens was detected in both groups after the challenge (week 0, Figures 1A–C). Animals of the EB group showed a peak-up response of IFN-γ to the three antigens at weeks 3 and 5, which was significantly higher than in animals of the IN group (*P* < 0.01; except *P* < 0.05 for PPD-B at week 5). IFN-γ-specific responses in intranasally challenged animals increased progressively over weeks, while IFN-γ levels elicited after the endobronchial challenge decreased at the end of the experiment when responses in both groups tended to converge (no significant differences at weeks 7 and 9).

**Figure 2:**
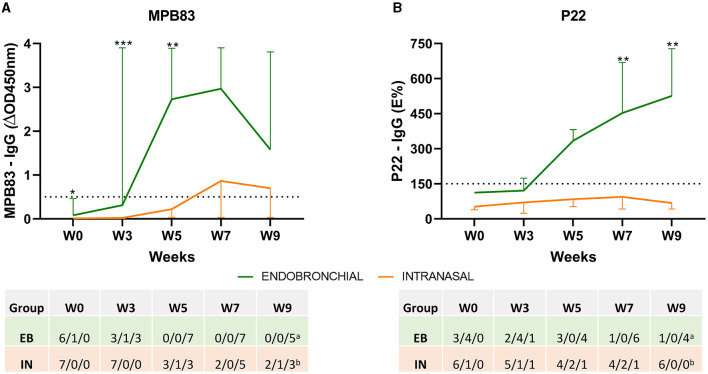
Antibody responses before and after the challenge. The figure shows the mean levels of IgG against MPB83 and P22 antigens measured by ELISA throughout the study. **(A)** MPB83-ELISA results are expressed as ΔOD450mn ± 95% CI. A sample was considered positive when ΔOD450nm ≥ 0.05. **(B)** P22-ELISA results are expressed as E%. E% = [mean OD450nm of the antigen-coated well/(2 × mean negative control OD450nm)] × 100. A sample is considered negative when it is ≤ 100%, inconclusive between 110 and 150% and seroconverted when the percentage is ≥150%. The dashed horizontal line shows the cutoff point for positivity. *P < 0.05, **P < 0.01, ***P < 0.001. Mann–Whitney test. The table indicates the numbers of animals and their seroconversion status: negative/inconclusive/positive. Groups: endobronchial (EB, *N* = 7, ^a^*N* = 5 at W9) and intranasal (IN, *N* = 7, ^b^*N* = 6 at W9).

IgG levels to the MPB83 antigen and the P22 antigenic complex, as well as individual seroconversion, were studied using two ELISAs throughout the study (Figure 2). Endobronchial challenge induced an early increase in MPB83 IgG-specific responses (a peak-up response at weeks 5 and 7), and all EB animals seroconverted at week 5 p.c. On the contrary, only a mild response was detected in IN animals, seroconversion was not detected in 2 of them throughout the experiment, and the other five animals seroconverted between weeks 5 and 9 (Figure 2A). In comparison with MPB83, IgG responses to P22 were more progressive in the EB group, reaching a peak at the end of the experiment (week 9), but seroconversion was detected in six of seven animals. IgG responses to P22 were practically undetectable in the IN group throughout the experiment, and only one animal showed a clear seroconversion (Figure 2B).

### 3.2. Disease-related clinical signs appeared later in intranasally challenged goats

**Figure 3:**
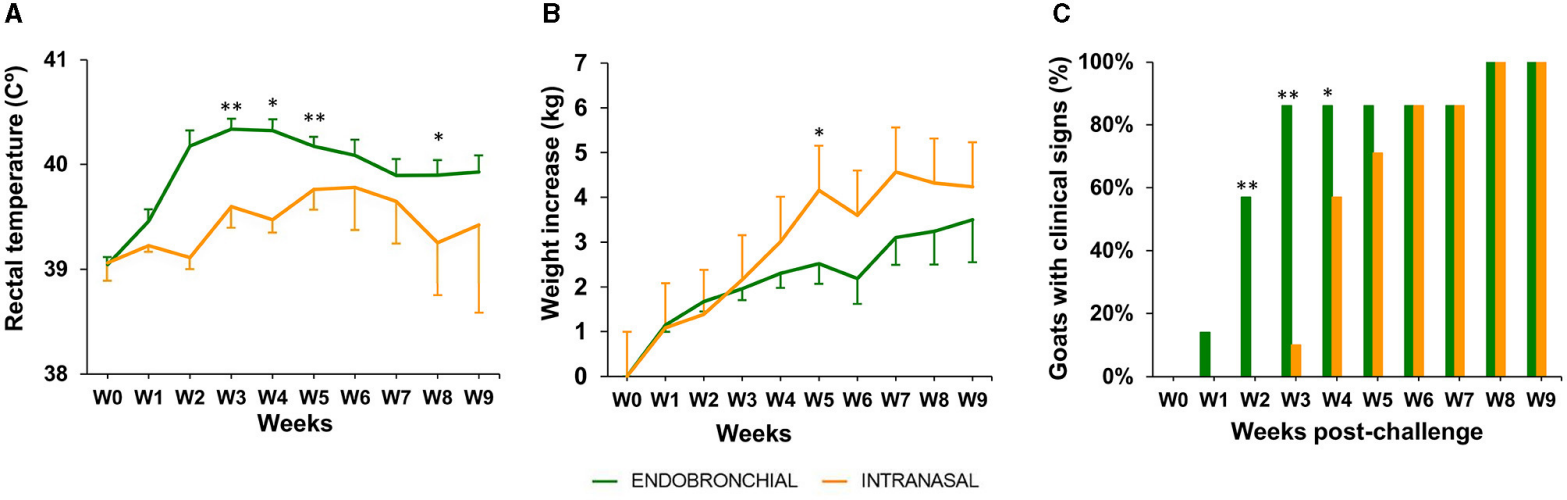
Rectal temperature, body weight, and clinical signs post-challenge. **(A)** The weekly mean rectal temperature of each group expressed in °C ± 95% CI. **(B)** Weekly mean cumulative body weight increases of each group expressed in ΔKg (Kg at each week post-challenge minus Kg at week 0) ± 95% CI. **(C)** The percentage of animals with the presence of TB-related clinical signs in each group. The clinical signs observed were cough, dyspnea, eye discharge, enlarged lymph nodes, nasal discharge, and anorexia (the groups were compared using the chi-square test). Groups: endobronchial (green, *N* = 7; *N* = 5 at weeks 8 and 9) intranasal (orange, *N* = 7; *N* = 6 at weeks 8 and 9). Body temperature and weight were compared using the one-way unpaired *t*-test. *P < 0.05, **P < 0.01.

Rectal temperature, body weight, and clinical signs of TB were weekly recorded throughout the experiment (Figure 3).

In the EB group, a peak of mean rectal temperature was observed between weeks 2 and 4 p.c., being statistically significantly higher compared with the IN group (*P* < 0.01, weeks 2 and 4; *P* < 0.05, week 3; Figure 3A). A mild peak was also observed in the IN group between weeks 5 and 7, although still remained slightly lower than the EB group, and decreased at week 8 to be significantly lower than the EB group again (*P* < 0.05).

The mean body weight gain was greater in the IN group compared with the EB group since week 4, being statistically significant at week 5 (P <0.05, Figure 3B), although a stagnation in body weight increase was observed from week 5 onward. In contrast, animals belonging to the EB group showed a mild increase in mean body weight from week 6 to the end of the experiment, where the difference in mean cumulative weight gain (ΔKg, Kg at weeks 9–0) between the IN and EB groups was minimal (4.2 ± 3.3 95% CI and 3.5 ± 2.9 95%CI, respectively).

The presence or absence of clinical signs, other than weight loss or fever, compatible with TB was recorded in all challenged animals until the end point of the experiment (Figure 3C). The observed signs included cough, dyspnea, eye discharge, enlarged lymph nodes, runny nose, and anorexia. An animal of the EB group showed clinical signs at week 1, 4 at week 2, and 6 from week 3 to the endpoint, whereas a 2-week delay in the onset of clinical signs was observed in the IN group: 1, 4, and 6 animals at weeks 3, 4, and from 5 to the endpoint, respectively (Figure 3C). The proportion of animals with clinical signs was significantly higher in the EB group at weeks 2 (*P* < 0.01), 3 (*P* < 0.01), and 4 (*P* < 0.05) compared with the IN group. All experimental animals showed clinical signs at weeks 8 and 9.

### 3.3. Endobronchially challenged animals showed more severe lung lesions

**Figure 4:**
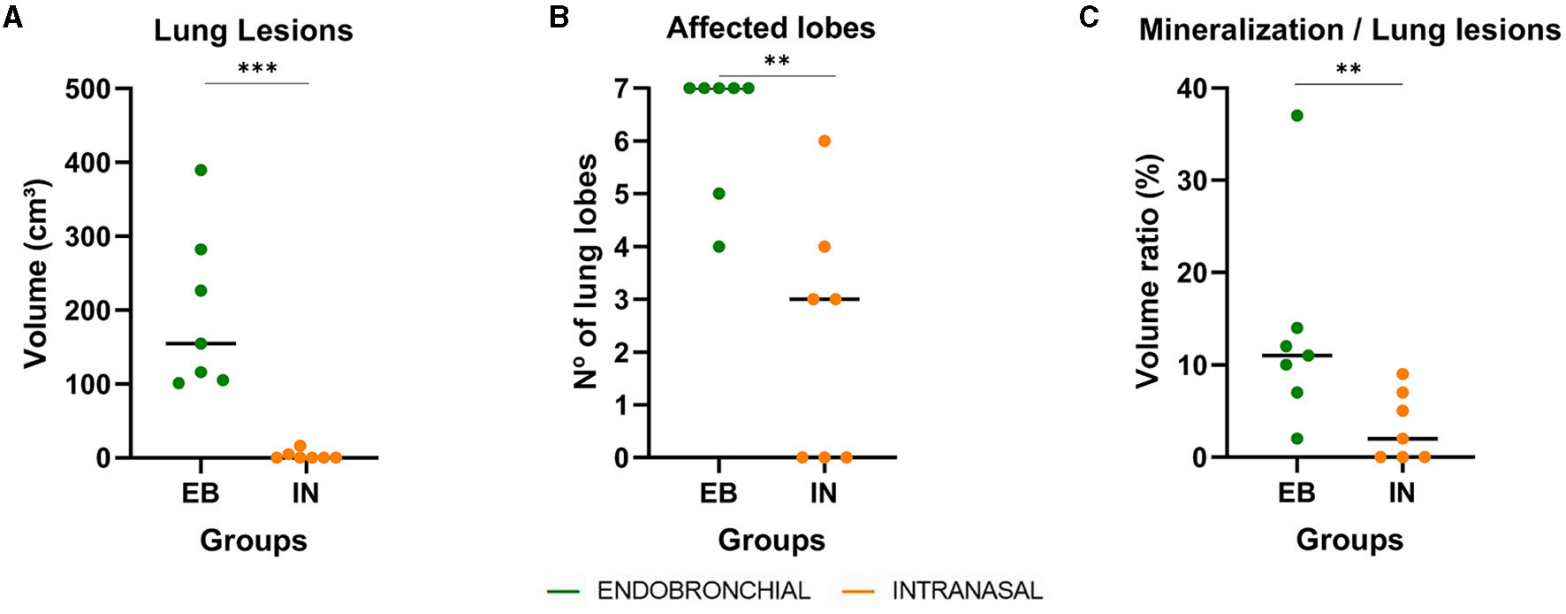
Lung pathological findings in endobronchially (EB) and intranasally (IN) *M. caprae-*challenged goats. **(A)** Volumes of lung TB lesions expressed in cm3 measured by computed tomography (CT) scan. **(B)** Number of lung lobes with TB lesions. **(C)** The ratio between the volume of mineralization and the volume of lung lesions expressed in %. Groups: endobronchial (*n* = 7, green) and intranasal (*n* = 7, orange). **P <0.01, ***P <0.001, Mann–Whitney test.

All goats showed TB lesions at necropsy. Lung lesions were quantitatively assessed by CT. All animals challenged by the EB route (7/7) had lung lesions, while only 4/7 animals of the IN group presented lung lesions, and the severity and dissemination of these lesions were very much lower compared with those found in the EB group. Figure 4 shows the extension of lung TB granulomas expressed as the volume of lung lesions, the intrapulmonary spread of TB lesions expressed as the number of affected lung lobes, and the degree of development of lung TB lesions expressed as the volume ratio between mineralization and lesion of each group. The EB group showed a significantly larger median volume of lung lesions compared with the IN group (*P* < 0.001, Figure 4A). In addition, the EB group showed a higher number of affected lobes compared with the IN group (*P* < 0.01, Figure 4B). Five out of seven EB animals showed TB lesions in all seven pulmonary lobes, while only one IN animal showed lesions in six pulmonary lobes. The EB group also showed a significantly greater median volume of lung mineralization (18.36 cm3, 95% CI: 2. 634–84.53, *P* < 0.001) compared with the IN group (0 cm3, 95% CI: 0–0.72), as well as higher median proportion of mineralization of lung lesions (*P* < 0.01; Figure 4C) indicative of a faster progression of pulmonary TB infection.

### 3.4. Intranasal challenge caused more severe lesions in the head but less generalized TB forms

**Figure 5:**
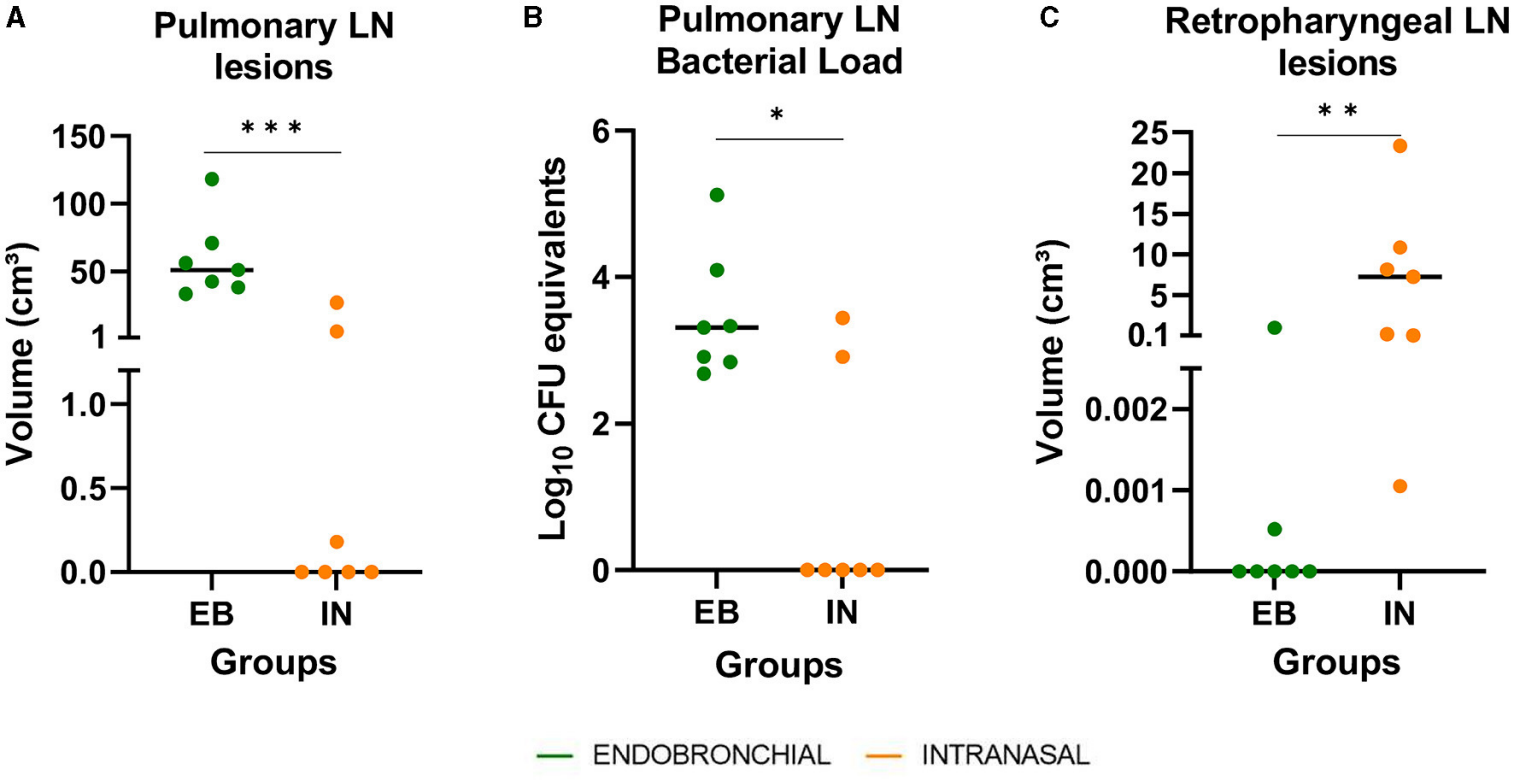
Post-mortem results in respiratory lymph nodes (LN). **(A)** The volume of lesions in pulmonary LN (tracheobronchial and mediastinal) expressed in cm3. **(B)** *M. caprae* DNA load in pulmonary LN, measured by quantitative PCR and expressed as Log_10_ CFU equivalents. **(C)** The volume of lesions in retropharyngeal LN expressed in cm3. Groups: endobronchial (EB, green)-challenged and intranasal (IN, orange)-challenged. *P < 0.05, **P < 0.01, ***P < 0.001, Mann–Whitney test.

All intranasally challenged animals showed lesions in retropharyngeal (RF) LNs, while these lesions were only detected in 2/7 animals of the EB group, and the median volume of lesions in the RF LNs was significantly higher in the IN group compared with the EB group (*P* < 0.01, Figure 5C). In contrast, only 3/7 animals that received the IN challenge developed TB lesions in pulmonary LNs, whereas these lesions were macroscopically found in all EB-challenged ones. The median volume of TB lesions in pulmonary LN was significantly higher in the EB group compared with the IN group (*P* < 0.001, Figure 5A), and the bacterial DNA load in pulmonary LNs, estimated as CFU equivalents by qPCR, was also significantly higher in the EB group (*P* < 0.05, Figure 5B).

**Figure 6:**
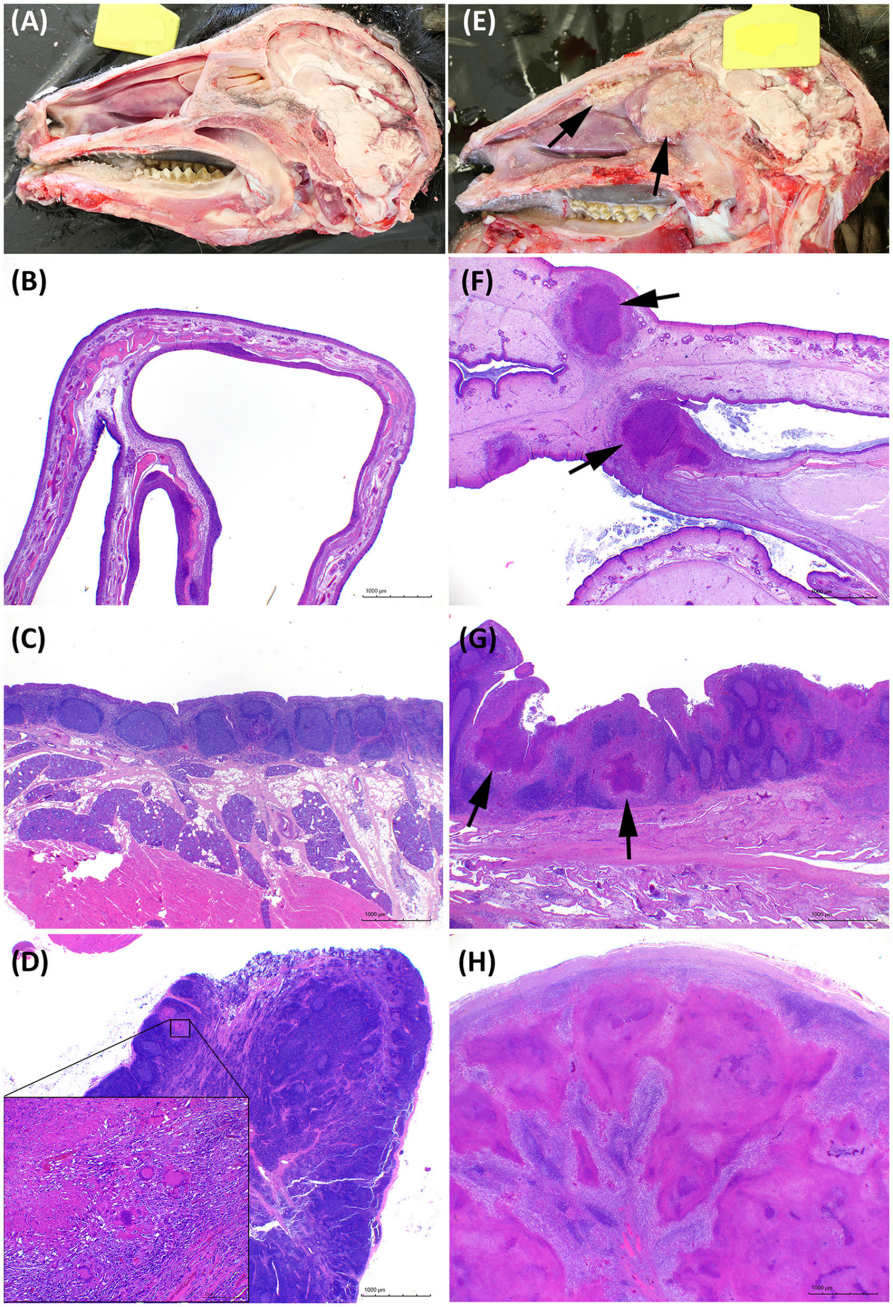
Pathological features in the head. **(A–D)** Representative images of goats challenged by the endobronchial route (EB). **(E–H)** Representative images of goats challenged by the intranasal route (IN). **(A, E)** Longitudinal section of the head. Granulomatous lesions are marked with arrows in the IN-challenged goat. **(B, F)** Transversal histological section of a normal nasal turbinate compared with an enlarged edematous nasal turbinate with multifocal granulomatous lesions (arrows). Hematoxylin and Eosin stain (H&E), bars 1,000 μm. **(C, G)** Nasopharyngeal mucosa with mucosal-associated lymphoid tissue. Noticeable multiple granulomas (arrows) in the IN animal in G. H&E, bars 1,000 μm. **(D, H)** Tuberculous lesions in the retropharyngeal lymph nodes of the EB-challenged group were absent or minimal as the one shown in the insert in **(B)**, or extensive and obliterating the whole lymph node in the IN group as shown in H. H&E, bars 1,000 μm.

All extrapulmonary lesions macroscopically compatible with TB were confirmed as such by histopathological examination. In general, the IN group presented major affectation of the head, whereas more severe TB-compatible lesions in the thoracic and abdominal cavities were found in the EB group. Four out of seven animals of the IN group had an intense granulomatous inflammatory infiltrate in the nasal cavity (see Figures 6E, F), and the three animals had lesions in the retropharyngeal and/or submandibular lymph nodes.

**Figure 7:**
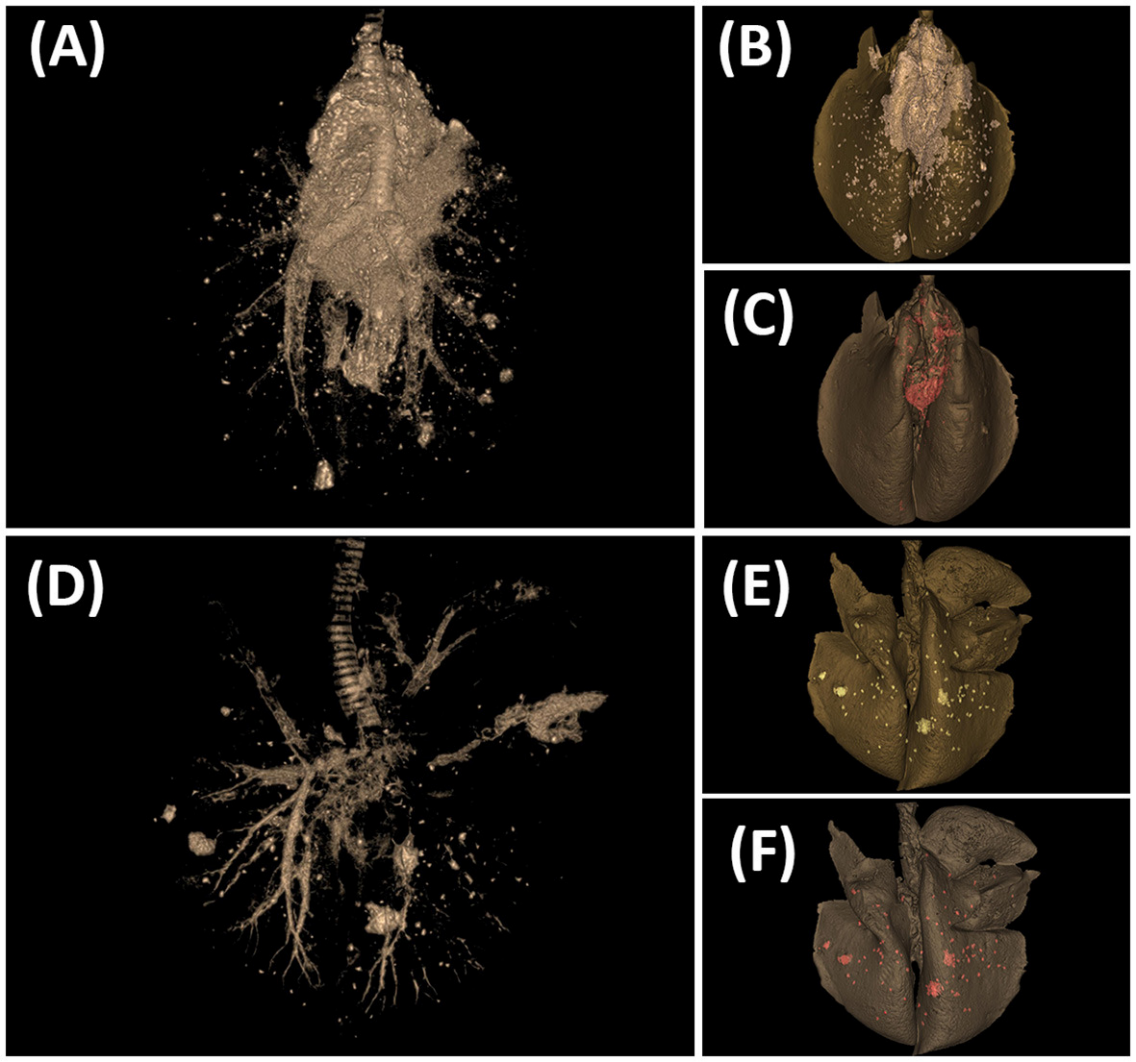
Tuberculosis lesions evidenced by computed tomography (CT) scan in the lungs. **(A–C)** Goat challenged by the endobronchial route. **(D–F)** Goat challenged by the intranasal route. **(A, D)** CT scan image showing the tracheobronchial tree and TB lesions. **(B, E)** CT-rendered image of TB lesions (whitish) on the total volume of the lung (dark gray). **(C, F)** CT-rendered image of mineralization (red) on the total volume of the lung (dark gray).

Figures 6, 7 show examples of TB lesions developed in representative IN- and EB-challenged animals in the head and lungs, respectively. The longitudinal section of the head of intranasally challenged goats evidenced bilateral extensive multifocal to coalescent granulomatous lesions in the nasal cavity of 4/7 goats; the endoturbinates and the middle nasal concha (on the caudal aspect of the nasal cavity) were always the most affected, while the dorsal and ventral nasal concha and the nasal septum were more variably involved (Figure 6E). Microscopically, granulomas of variable extension were observed in the nasal turbinates (Figure 6F) and nasopharyngeal mucosa (Figure 6G), while the EB-challenged animals did not present visible lesions in this localization (Figures 6A, B). Lesions in the head lymph nodes were more severe in the IN group (Figure 6H) while minimal or absent in the EB group (Figure 6D). Microscopically, the lesional patterns observed were equivalent in both groups, differing only in their extension and location. On the other hand, the rendered images of the lungs obtained by CT disclosed more extensive lung lesions and a higher total volume of lung mineralization in the EB-challenged animals (Figure 7).

**Figure 8:**
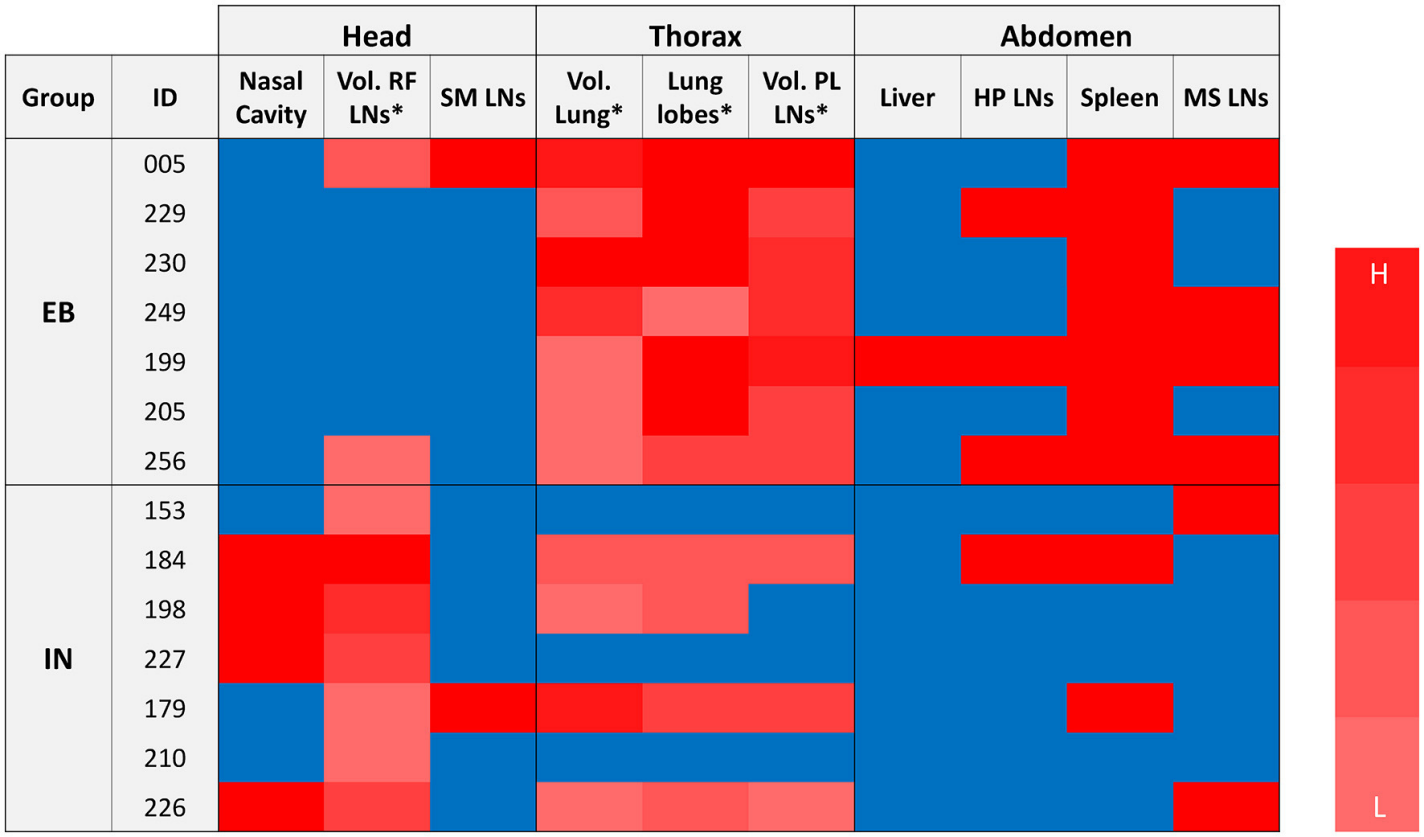
Distribution of TB lesions. Blue and red cells indicate the absence and presence of lesions, respectively. (*) In the case of RF LN, pulmonary LN, and lungs, a gradation of red levels represents the quintile distribution of volumes of lesions or number of affected lobes (scale: L -Low: 1- to H -High: 5-). Groups: endobronchial (EB)-challenged (*N* = 7) and intranasal (IN)-challenged (*N* = 7). LN, lymph node; SM, submandibular; RF, retropharyngeal; PL, pulmonary; HP, hepatic; MS, mesenteric.

Figure 8 presents the distribution of TB lesions in all the animals of each group. The animals of the EB group have minimal involvement in the head structure whereas all animals of the IN group had lesions in the head (all of them in the retropharyngeal LNs). On the other hand, all animals of the EB group had lesions in the thorax but also in the abdominal cavity (especially in the spleen), indicative of early hematogenous generalization.

## 4. Discussion

Since vaccination of cattle against TB is currently forbidden in the EU due to its interference with the current diagnostic tools used in eradication programs, the goat represents an excellent translational model to investigate the effect of vaccines against TB, which can be tested under field conditions (Pérez de Val et al., 2012, 2013; Bezos et al., 2017; Arrieta-Villegas et al., 2018; Roy et al., 2018; Melgarejo et al., 2022). Additionally, goats are also a suitable animal model for human TB because they resemble more closely than small laboratory animals and possess key anatomical features such as the complex bronchial tree (Mclaughlin et al., 1961) and lung parenchyma organized in a net of interlobular septa that tend to encapsulate the lesions (Gil et al., 2010), the respiratory tract and lung vascularization (Magno et al., 1990), and the respiratory lymphoid tissues (Liebler-Tenorio and Pabst, 2006).

The present study aimed to evaluate the IN nebulization of *M. caprae* as a field-like infection entrance pathway. The rationale was to better reproduce the features of the natural progression of TB infection but in a controlled manner, in order to improve the monitoring of clinical, immunological, bacteriological, and pathological parameters that could be used as predictors of vaccine efficacy in further studies. The new approach was compared with the well-established experimental infection through endobronchial inoculation (Pérez de Val et al., 2011). The severity and the progression of the disease were not only compared by the different distribution of lesions in the EB and IN groups but also they were holistically evaluated considering all the parameters mentioned above, according to previous investigations on TB pathogenicity in goats (Pérez de Val et al., 2013; Domingo et al., 2014).

The results showed that IN infection slowed the progression of the disease in comparison with the EB route in terms of cell-mediated immune response kinetics (antigen-specific IFN-γ responses) and the onset of clinical signs (body weight gain, rectal temperature, and respiratory signs). The delay of proinflammatory responses (determined by IFN-γ levels) in the IN group may be explained by a slower development of granulomatous lesions that normally show a direct correlation with antigen-specific IFN-γ responses (Vordermeier et al., 2002; Pérez de Val et al., 2012). The kinetics and intensity of proinflammatory responses in the IN group are consistent with the more progressive, less intense, and delayed rectal temperature peaks (weeks 5–7 p.c in the IN group instead of weeks 2–4 p.c. in the EB group). The delayed seroconversion rates (MPB83) or absence of detectable humoral responses (P22) in most animals of the IN group are also indicative of a slower progression of the disease. The lower intensity of MPB83-specific IgG responses in the IN group can also be associated with a lower severity of TB lesions and bacterial burden, as previously described in cattle and goats (Lyashchenko et al., 2004; Pérez de Val et al., 2011).

However, at the end of the study, all IN-challenged animals showed TB-compatible clinical signs indicating that the infection was well-established. This was also supported by the fact that the cumulative body weight gains of the IN and EB groups tended to converge at the end of the experiment. Previous studies, carried out by our research group, demonstrated that body weight increase in goat kids, experimentally challenged with *M. caprae*, inversely correlates with pathological TB outcome (Melgarejo et al., 2022).

From the pathological point of view, the EB infection induces faster disease progression with the early development of large-extensive pulmonary lesions similar to those found in certain chronic advanced cases of caprine TB in the field (Domingo et al., 2014). In contrast, after 9 weeks, goats infected through the IN route showed moderate pulmonary lesions similar to those usually obtained in natural infections, characterized by small to medium, multifocal lung nodular granulomatous lesions with a varying degree of encapsulation, variable presence of satellite granulomas, and occasionally, bigger coalescing lesions with central cavitation. These lung lesions are often associated with lesions in the thoracic lymph nodes (Domingo et al., 2014). The main drawback of the IN route is the intense granulomatous lesions found in the nasal cavity and head LN in some animals, which were artificially induced by the nebulization platform used to inoculate the mycobacteria. These lesions are not usually observed in field cases (Sanchez et al., 2011). This might be explained by the fact that during the challenge, a considerable proportion of the droplets containing mycobacteria bulk delivered into the nasal cavity was not inhaled and remained in the upper airways, thus generating local granulomatous lesions. In contrast, airborne aerosols transporting mycobacteria in field conditions are likely to access the lung parenchyma directly and in discrete installments, thus bypassing the upper airways (Daniel et al., 2009).

In an experimental infection conducted in calves inoculated intranasally with *M. bovis*, most animals (11/12) developed TB granulomatous lesions in LNs associated with the upper respiratory tract, and only four of them had lesions in the lungs or tracheobronchial LN (McCorry et al., 2005). In the present study, we investigated not only the head LNs but also performed a longitudinal section of the skull to investigate the nasal cavity. We found macroscopic intranasal granulomas in 4/7 animals that partially obstructed the upper airways and, in one case, provoked severe dyspnea that led to the humane euthanasia of one animal at week 8 before the completion of the experiment. Putative variations of the location of the cannula in the dorsal or middle nasal meatus vs. the ventral nasal meatus could explain the location of lesions in the caudal aspect of the nasal cavity in 4/7 animals, while in the 3/7 animals with no lesions, the cannula was likely introduced in the ventral meatus. In one of these animals, the lesions in the lung were the most severe in the group (animal 179, suggestive of an effective delivery of the inoculum to the lung), while the other two only developed lesions in the retropharyngeal lymph nodes. In addition, two animals of the IN group had lesions in the mesenteric LN, and this could be explained by the partial ingestion of the inoculum (in the case of animal 153, no lesion was observed in the nose), by swallowing the mycobacteria from the granulomatous lesions developing in the nasal cavity and/or associated lymph nodes (in the case of animal 226) or, less likely, due to lymphohematogenous dissemination to the abdominal cavity. The involvement of mesenteric LN was a frequent finding in natural settings in a field study carried out in a goat herd, where the most likely transmission pathway was the shared feeders between infected and susceptible animals (Vidal et al., 2017). Spread of TB lesions beyond the upper airways and thoracic cavity also occurred in all EB-challenged animals (i.e., all of them showed lesions in the spleen), most likely due to a lymphohematogenous spreading from the primary complex generated in the lungs and pulmonary LN, similar to that reported in previous studies (Arrieta-Villegas et al., 2018; Melgarejo et al., 2022). This phenomenon could also have occurred in two IN-challenged animals that showed lesions in the spleen (suggestive of hematogenous dissemination), one of which had the most extensive lung TB lesions in the IN-challenged group, without nasal cavity affectation.

To properly develop a suitable infection model to measure the efficacy of TB treatments, it would be necessary to refine the nebulization procedure to ensure that most of the inoculum enters into the trachea, avoiding the biased involvement of the nasal cavity. This could be accomplished by introducing the cannula directly into the ventral nasal meatus. Another explanation for the skewed distribution of lesions in the IN-challenged goats could be the fact that the animals might not have inhaled normally when inoculated and even tried to reject or swallow the inoculum. Thus, sedation of the goats before the experimental challenge could also facilitate a more direct delivery to the lungs.

To date, different approaches to MTBC experimental infections have been modelized in goats: transthoracic (Bezos et al., 2010, 2015), endobronchial (Pérez de Val et al., 2011; Wedlich et al., 2022), aerosolization (Gonzalez-Juarrero et al., 2013), and the infection by contact approach, i.e., the exposure of susceptible goats to infected ones by close contact (Bezos et al., 2017; Roy et al., 2019). The latter has the advantage to mimic natural infection in the herd but has the disadvantage of not controlling critical points such as the infection time point, infection dose, and infection route, which hinder the monitorization of the infection progress and, most critically, comparison between treatment groups. Similarly, the use of aerosolizers enables the inhalation of mycobacteria (Gonzalez-Juarrero et al., 2013) but does not allow to deposit the inoculum in a defined site of the respiratory tract at the start-point of the experiment, to compare the pathological evolution from the infection site among the treatment groups. The atomizing device used in the present study sprays droplets > 30 mm in diameter that resemble those excreted by sneezing or coughing. With the IN nebulization one can control the infection time point and infection dose conditions but still not ensure the infection site within the respiratory tract, resulting in moderate pulmonary TB lesions in only 4/7 animals, probably because a significant fraction of the inoculum was retained in the nasal cavity, whereas all goats infected by EB inoculation showed pulmonary TB lesions as previously described in all experimental infections of goats with *M. caprae* or *M. bovis* by this route (Pérez de Val et al., 2011, 2012, 2013; Arrieta-Villegas et al., 2018, 2020; Melgarejo et al., 2022; Wedlich et al., 2022). However, the EB model, as does the transthoracic one, can induce advanced caseous-necrotizing lesions even in vaccinated individuals, limiting the capacity to predict the protective effect of vaccinated candidates (Arrieta-Villegas et al., 2020). In contrast, the IN infection induced low-to-moderate pulmonary lesions with a low degree of mineralization, suggesting a more initial developmental stage that may facilitate the evaluation of the containment of TB lesions spreading in immunized animals. Optimization of the model is required to reach a compromise among (A) conditions control (dose, inoculation point), (B) duration of the experiment, and (C) reproducibility of TB field phenotype in order to precisely evaluate the efficacy of the tested vaccines.

## Data availability statement

The original contributions presented in the study are included in the article/supplementary material, further inquiries can be directed to the corresponding authors.

## Ethics statement

The animal studies were approved by Animal Welfare Committee of the Generalitat de Catalunya. The studies were conducted in accordance with the local legislation and institutional requirements. Written informed consent was not obtained from the owners for the participation of their animals in this study because the animals were purchased for these studies.

## Author contributions

BP and EV conceived and planned the study. CM, AC, CP, MM, ZC, GC, XM, YE, MD, EV, and BP performed the experiments and contributed to data collection. CM, AC, JF, CP, YE, EV, and BP analyzed and interpreted the data. BP and MD acquired funding and were in charge of project administration. CM, EV, and BP wrote the original draft. MD contributed to writing, reviewing, and editing the final version of the manuscript. All authors contributed to the article and approved the submitted version.

## References

[B1] Aranaz A. Cousins D. Mateos A. Domínguez L. (2003). Elevation of *Mycobacterium tuberculosis* subsp. caprae Aranaz et al. 1999 to species rank as *Mycobacterium caprae* comb. nov., sp. nov. Int. J. Syst. Evol. Microbiol. 53, 1785–1789. 10.1099/ijs.0.02532-0 14657105

[B2] Arrieta-Villegas C. Allepuz A. Grasa M. Martín M. Cervera Z. Mercader I.. (2020). Long-term efficacy of BCG vaccination in goat herds with a high prevalence of tuberculosis. Sci. Rep. 10, 20369. 10.1038/s41598-020-77334-1 33230112 PMC7683592

[B3] Arrieta-Villegas C. Perálvarez T. Vidal E. Puighibet Z. Moll X. Canturri A.. (2018). Efficacy of parenteral vaccination against tuberculosis with heat-inactivated *Mycobacterium bovis* in experimentally challenged goats. PLoS ONE 13, e0196948. 10.1371/journal.pone.0196948 29742150 PMC5942842

[B4] Balseiro A. Altuzarra R. Vidal E. Moll X. Espada Y. Sevilla I. A.. (2017). Assessment of BCG and inactivated *Mycobacterium bovis* vaccines in an experimental tuberculosis infection model in sheep. PLoS ONE 12, e0180546. 10.1371/journal.pone.0180546 28678885 PMC5498051

[B5] Bezos J. Casal C. Álvarez J. Roy A. Romero B. Rodríguez-Bertos A.. (2017). Evaluation of the *Mycobacterium tuberculosis* SO2 vaccine using a natural tuberculosis infection model in goats. Vet. J. 223, 60–67. 10.1016/j.tvjl.2017.04.006 28671074

[B6] Bezos J. Casal C. Díez-Delgado I. Romero B. Liandris E. Álvarez J.. (2015). Goats challenged with different members of the *Mycobacterium tuberculosis* complex display different clinical pictures. Vet. Immunol. Immunopathol. 167, 185–189. 10.1016/j.vetimm.2015.07.009 26235598

[B7] Bezos J. de Juan L. Romero B. Álvarez J. Mazzucchelli F. Mateos A.. (2010). Experimental infection with *Mycobacterium caprae* in goats and evaluation of immunological status in tuberculosis and paratuberculosis co-infected animals. Vet. Immunol. Immunopathol. 133, 269–275. 10.1016/j.vetimm.2009.07.018 19716181

[B8] Buddle B. M. Martin Vordermeier H. Hewinson R. G. (2016). Experimental infection models of tuberculosis in domestic livestock. Microbiol. Spectr. 4, 44–66. 10.1128/microbiolspec.TBTB2-0017-2016 27726786

[B9] Cano-Terriza D. Risalde M. A. Rodríguez-Hernández P. Napp S. Fernández-Morente M. Moreno I.. (2018). Epidemiological surveillance of *Mycobacterium tuberculosis* complex in extensively raised pigs in the south of Spain. Prev. Vet. Med. 159, 87–91. 10.1016/j.prevetmed.2018.08.015 30314795

[B10] Català M. Prats C. López D. Cardona P. J. Alonso S. (2020). A reaction-diffusion model to understand granulomas formation inside secondary lobule during tuberculosis infection. PLoS ONE 15, 1–20. 10.1371/journal.pone.0239289 32936814 PMC7494083

[B11] Daniel R. Evans H. Rolfe S. De La Rua-Domenech R. Crawshaw T. Higgins R. J.. (2009). Papers: outbreak of tuberculosis caused by *Mycobacterium bovis* in golden Guernsey goats in great britain. Vet. Rec. 165, 335–342. 10.1136/vr.165.12.335 19767636

[B12] Domingo M. Vidal E. Marco A. (2014). Pathology of bovine tuberculosis. Res. Vet. Sci. 97, S20–S29. 10.1016/j.rvsc.2014.03.017 24731532

[B13] Gil O. Díaz I. Vilaplana C. Tapia G. Díaz J. Fort M.. (2010). Granuloma encapsulation is a key factor for containing tuberculosis infection in minipigs. PLoS ONE 5, e10030. 10.1371/journal.pone.0010030 20386605 PMC2850319

[B14] Gonzalez-Juarrero M. Bosco-Lauth A. Podell B. Soffler C. Brooks E. Izzo A.. (2013). Experimental aerosol *Mycobacterium bovis* model of infection in goats. Tuberculosis 93, 558–564. 10.1016/j.tube.2013.05.006 23850102

[B15] Infantes-Lorenzo J. A. Moreno I. Risalde M. D. L. Á. Roy Á. Villar M. Romero B.. (2017). Proteomic characterisation of bovine and avian purified protein derivatives and identification of specific antigens for serodiagnosis of bovine tuberculosis. Clin. Proteom. 14, 36. 10.1186/s12014-017-9171-z 29142508 PMC5669029

[B16] Infantes-Lorenzo J. A. Moreno I. Roy A. Risalde M. A. Balseiro A. de Juan L.. (2019). Specificity of serological test for detection of tuberculosis in cattle, goats, sheep and pigs under different epidemiological situations. BMC Vet. Res. 15, 70. 10.1186/s12917-019-1814-z 30823881 PMC6397464

[B17] Liebler-Tenorio E. Pabst R. (2006). MALT structure and function in farm animals. Vet. Res. 37, 257–280. 10.1051/vetres:2006001 16611547

[B18] Lyashchenko K. Whelan A. O. Greenwald R. Pollock J. M. Andersen P. Hewinson R. G.. (2004). Association of tuberculin-boosted antibody responses with pathology and cell-mediated immunity in cattle vaccinated with *Mycobacterium bovis* BCG and infected with M. bovis. Infect. Immun. 72, 2462–2467. 10.1128/IAI.72.5.2462-2467.2004 15102752 PMC387859

[B19] Magno M. Long Nadel J. Wanner A. Lockhart A. (1990). Comparative anatomy of the tracheobronchial circulation. Eur. Respir. J. 13, 557S−563S. 2127528

[B20] McCorry T. Whelan A. O. Welsh M. D. McNair J. Walton E. Bryson D. G.. (2005). Shedding of *Mycobacterium bovis* in the nasal mucus of cattle infected experimentally with tuberculosis by the intranasal and intratracheal routes. Vet. Rec. 157, 613–618. 10.1136/vr.157.20.613 16284329

[B21] Mclaughlin R. F. Tyler W. S. Canada R. (1961). A study of the subgross pulmonary anatomy in various mammals. Am. J. Anat. 108, 149–165. 10.1002/aja.1001080203

[B22] Melgarejo C. Planas C. Cobos A. Arrieta-Villegas C. Sevilla I. A. Bezos J.. (2022). A proof-of-conclept study to investigate the efficacy of heat-inactivated autovaccines in *Mycobacterium caprae* experimentally challenged goats. Sci. Rep. 12, 22132. 10.1038/s41598-022-26683-0 36550177 PMC9780325

[B23] Napp S. Allepuz A. Mercader I. Nofrarias M. Lopez-Soria S. Domingo M.. (2013). Evidence of goats acting as domestic reservoirs of bovine tuberculosis. Vet. Rec. 172, 663. 10.1136/vr.101347 23687108

[B24] Pérez de Val B. López-Soria S. Nofrarías M. Martín M. Vordermeier H. M. Villarreal-Ramos B.. (2011). Experimental model of tuberculosis in the domestic goat after endobronchial infection with *Mycobacterium caprae*. Clin. Vacc. Immunol. 18, 1872–1881. 10.1128/CVI.05323-11 21880849 PMC3209027

[B25] Pérez de Val B. Napp S. Velarde R. Lavín S. Cervera Z. Singh M.. (2017). serological follow-up of tuberculosis in a wild boar population in contact with infected cattle. Transbound. Emerg. Dis. 64, 275–283. 10.1111/tbed.12368 25944524

[B26] Pérez de Val B. Vidal E. Villarreal-Ramos B. Gilbert S. C. Andaluz A. Moll X.. (2013). A multi-antigenic adenoviral-vectored vaccine improves BCG-induced protection of goats against pulmonary tuberculosis infection and prevents disease progression. PLoS ONE 8, e81317. 10.1371/journal.pone.0081317 24278420 PMC3836889

[B27] Pérez de Val B. Villarreal-Ramos B. Nofrarías M. López-Soria S. Romera N. Singh M.. (2012). Goats primed with *Mycobacterium bovis* BCG and boosted with a recombinant adenovirus expressing Ag85A show enhanced protection against tuberculosis. Clin. Vacc. Immunol. 19, 1339–1347. 10.1128/CVI.00275-12 22761299 PMC3428397

[B28] Ramirez I. C. Santillan M. A. Dante V. (2003). The goat as an experimental ruminant model for tuberculosis infection. Small Rumin. Res. 47, 113–116. 10.1016/S0921-4488(02)00243-228671074

[B29] Rodríguez S. Bezos J. Romero B. de Juan L. Álvarez J. Castellanos E.. (2011). *Mycobacterium caprae* infection in livestock and wildlife, Spain. Emerg. Infect. Dis. 17, 532–535. 10.3201/eid1703.100618 21392452 PMC3165998

[B30] Roy Á. Risalde M. A. Bezos J. Casal C. Romero B. Sevilla I.. (2018). Response of goats to intramuscular vaccination with heat-killed Mycobacterium bovis and natural challenge. Comp. Immunol. Microbiol. Infect. Dis. 60, 28–34. 10.1016/j.cimid.2018.09.006 30396427

[B31] Roy A. Tomé I. Romero B. Lorente-Leal V. Infantes-Lorenzo J. A. Domínguez M.. (2019). Evaluation of the immunogenicity and efficacy of BCG and MTBVAC vaccines using a natural transmission model of tuberculosis. Vet. Res. 50, 1–3. 10.1186/s13567-019-0702-7 31615555 PMC6792192

[B32] Sanchez J. Tomás L. Ortega N. Buendía A. J. del Rio L. Salinas J.. (2011). Microscopical and immunological features of tuberculoid granulomata and cavitary pulmonary tuberculosis in naturally infected goats. J. Comp. Pathol. 145, 107–117. 10.1016/j.jcpa.2010.12.006 21334000

[B33] Seva J. Menchén V. Navarro J. A. Pallarés F. J. Villar D. Vásquez F.. (2002). Caprine tuberculosis eradication program: an immunohistochemical study. Small Rumin. Res. 46, 107–114. 10.1016/S0921-4488(02)00174-8

[B34] Tanner R. O'Shea M. K. Fletcher H. A. McShane H. (2016). *In vitro* mycobacterial growth inhibition assays: a tool for the assessment of protective immunity and evaluation of tuberculosis vaccine efficacy. Vaccine 34, 4656–4665. 10.1016/j.vaccine.2016.07.058 27527814

[B35] Vidal E. Arrieta-Villegas C. Grasa M. Mercader I. Domingo M. Pérez de Val B. (2017). Field evaluation of the efficacy of Mycobacterium bovis BCG vaccine against tuberculosis in goats. BMC Vet. Res. 13, 252. 10.1186/s12917-017-1182-5 28818102 PMC5561642

[B36] Vidal E. Burgaya J. Michelet L. Arrieta-Villegas C. Cantero G. de Cruz K.. (2022). Experimental *Mycobacterium microti* infection in bank voles (*Myodes glareolus*). *Microorganisms* 10, 135. 10.3390/microorganisms10010135 35056584 PMC8779978

[B37] Vidal E. Grasa M. Perálvarez T. Martín M. Mercader I. Pérez de Val B. (2018). Transmission of tuberculosis caused by *Mycobacterium caprae* between dairy sheep and goats. Small Rumin. Res. 158, 22–25. 10.1016/j.smallrumres.2017.11.01018665779

[B38] Vordermeier H. M. Chambers M. A. Cockle P. J. Whelan A. O. Simmons J. Hewinson R. G. (2002). Correlation of ESAT-6-specific gamma interferon production with pathology in cattle following *Mycobacterium bovis* BCG vaccination against experimental bovine tuberculosis. Infect. Immun. 70, 3026–3032. 10.1128/IAI.70.6.3026-3032.2002 12010994 PMC128013

[B39] Wedlich N. Figl J. Liebler-Tenorio E. M. Köhler H. von Pückler K. Rissmann M.. (2022). Video endoscopy-guided intrabronchial spray inoculation of *Mycobacterium bovis* in goats and comparative assessment of lung lesions with various imaging methods. Front. Vet. Sci. 9, 877322. 10.3389/fvets.2022.877322 35591868 PMC9113525

